# Boosting with variant-matched vaccines: an opportunity to win the race against Omicron

**DOI:** 10.1038/s41392-022-01049-0

**Published:** 2022-06-16

**Authors:** Jiayu Wang, Tianxia Lan, Qiu Sun

**Affiliations:** State Key Laboratory of Biotherapy, Cancer Center, West China Hospital, Sichuan University and Collaborative Innovation Center for Biotherapy, 610041 Chengdu, China

**Keywords:** Immunology, Biotechnology

Recently, Baoling Ying et al. published a study in *Cell* that investigated whether using BA.1-matched or historical mRNA vaccine as a booster could enhance the protection against Omicron infection.^1^ In this study, the team evaluated the anti-Omicron efficacies of these two types of vaccines as primary (two-dose) immunization series or third-dose boosters in mice, respectively (Fig. 1).

**Fig. 1:**
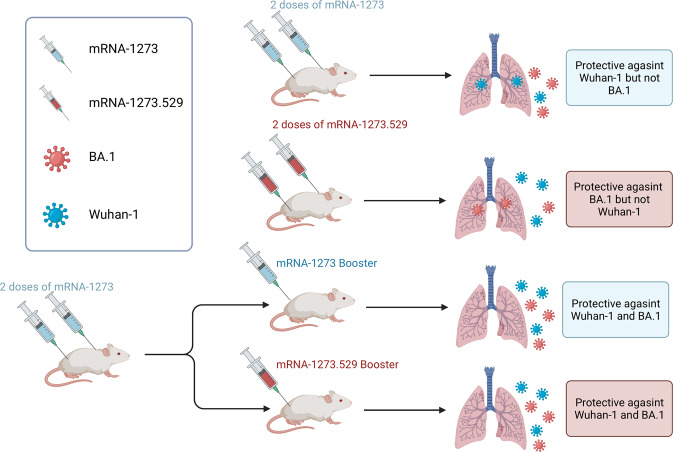
The protective activities of mRNA-1273 and mRNA-1273.529 as primary (two-dose) immunization series or third-dose boosters were evaluated in mice challenged with Wuhan-1 or BA.1

Many of the current outbreaks of COVID-19 around the world have been shown to be driven by the Omicron variant of SARS-CoV-2.^2^ Although vaccination remains one of the most efficient approaches to containing the pandemic, amino acid substitutions, deletions, and insertions in the spike protein of the Omicron variant have substantially impaired the efficacy of most licensed vaccines.^3^ Hence, more effective vaccines or vaccination strategies against Omicron are urgently needed.

To evaluate antibody responses elicited by the existing vaccine, IgG responses against Wuhan-1 (wild type) and BA.1 (Omicron variant) spike proteins induced by different doses of a preclinical version of mRNA-1273 were assessed by enzyme-linked immunosorbent assay (ELISA). The results suggest that although a high dose (5 μg) of mRNA-1273 could elicit a stronger antibody response against the spike protein and RBD of both Wuhan-1 and BA.1 than that elicited by the lower dose (0.1 μg), the IgG response against BA.1 was strikingly reduced compared to that against Wuhan-1 in both doses. The functional antibody responses were then characterized by focus-reduction neutralization test (FRNT). It was shown that serum from higher dose group has a stronger inhibitory effect on the infectivity of both Wuhan-1 and BA.1. Notably, the anti-infection capacity against BA.1 was lower than that against Wuhan-1.

Then, to assess the protective activity of mRNA-1273 as primary doses, the mice were immunized by two doses of 5 μg or 0.1 μg mRNA-1273 and then challenged with Wuhan-1 D614G or BA.1 virus. As the results show, comparing to the control RNA, mRNA-1273 reduced the viral loads of both Wuhan-1 D614G and BA.1 within nasal wash, nasal turbinate and lung. However, the vaccine-mediated attenuation of viral burden was less effective for BA.1. Notably, a high frequency of breakthrough infections was found in the low-dose group after the BA.1 challenge. In addition, only high-dose mRNA-1273 protected against the pathological changes as well as the upregulated expression of pro-inflammatory cytokines and chemokines associated with BA.1 infection.

To appraise the efficacy of mRNA-1273 as a booster, the K18-hACE2 mice received high (5 μg) or low (0.25 μg) doses of mRNA-1273 after the primary series of immunization. It was found that, comparing to non-boosted mice, the mice that received the third dose of mRNA-1273 had a higher level of serum antibody against both Wuhan-1 D614G and BA.1. Nonetheless, the mRNA-1273 booster was unable to completely prevent breakthrough infection caused by BA.1 in lungs, although it potently reduced the viral load of BA.1 in the respiratory tract.

To develop an alternative to the third dose of mRNA-1273, the team designed and produced a mRNA vaccine encoding the SARS-CoV-2 spike from the BA.1 virus, namely, mRNA-1273.529. Mice were immunized twice at 3-week intervals with 1 or 0.1 μg of mRNA-1273 or mRNA-1273.529 vaccines. Serum samples were collected on day 21 and day 36 for the assessment of serum antibody responses and neutralizing activity. The ELISA analysis revealed that the antibody responses against both Wuhan-1 spike and BA.1 spike induced by mRNA-1273 and mRNA-1273.529 in a dose-dependent manner, and they were stronger in day 36 than those in day 21. Additionally, it was found that the level of serum antibody binding to BA.1 RBD induced by mRNA-1273.529 was substantially higher than that induced by mRNA-1273. However, the pseudo-virus neutralization assay results suggest that two doses of 1 μg mRNA-1273.529 confer a high level of neutralizing serum antibodies against Omicron variants but a limited level of neutralizing serum antibodies against Wuhan-1 D614G, B.1.351 (Beta), or B.1.617.2 (Delta).

Moreover, to evaluate the antibody response elicited by mRNA-1273.529 when used as a booster dose, mice immunized by two doses of mRNA-1273 or control mRNA at 3-week intervals received 1 μg of control mRNA, mRNA-1273, or mRNA-1273.529 booster on day 98/99. Although both mRNA-1273.529 and mRNA-1273 boosters enhanced serum antibody neutralizing titers against the Omicron subvariants BA.1 and BA.2, the antibody response induced by the BA.1-matched mRNA-1273.529 was stronger. Additionally, to investigate the protective activity of mRNA-1273.529 as a booster dose, mice boosted with 1 μg of control mRNA, mRNA-1273, or mRNA-1273.529 were challenged with Wuhan-1 or BA.1. Then the viral loads in the nasal wash, the nasal turbinates, and the lungs were assessed by measuring viral RNA levels. Mice immunized with either a high (5 μg) or low (0.25 μg) dose of mRNA-1273 and boosted with mRNA-1273.529 showed almost complete protection against Wuhan-1 and BA.1. Nevertheless, mice immunized with low doses of primary immunization series and boosted with mRNA-1273 showed relatively higher BA.1 viral loads in nasal tubinates and lung, compared to those boosted by mRNA-1273.529. Furthermore, the cytokine and chemokine levels in lung homogenates were analyzed using cytokine/chemokine array. Interestingly, in mice vaccinated by control mRNA, Wuhan-1 induced stronger pro-inflammatory cytokine/chemokine responses than BA.1, suggesting the lower pathogenicity of Omicron variants.^4^ Mice immunized by 5 μg doses of primary immunizations and then boosted by either mRNA-1273 or mRNA-1273.529 showed substantially lower proinflammatory cytokine and chemokine responses induced by Wuhan-1 or BA.1 compared to the mice received control mRNA. However, for mice that received 0.25 μg primary immunizations, the protective activity of the mRNA-1273 booster against BA.1-induced lung inflammation was lower than that of mRNA-1273.529.

In summary, this study indicates that the efficacy of BA.1-matched mRNA-1273.529 against Omicron variants is higher than that against Wuhan-1, Beta and Delta variants. In addition, the protective activities against both Wuhan-1 and BA.1 of mRNA-1273.529 and the historical mRNA-1273 as boosters were significantly higher than the primary immunization series, where mRNA-1273.529 booster manifested stronger efficacy and immunogenicity against BA.1. This work brought to light the prospect and possibility of developing a clinically applicable Omicron-matched mRNA vaccine as a potential solution to the difficulties caused by the rapid surge of Omicron.

## References

[1] Ying B (2022). Boosting with variant-matched or historical mRNA vaccines protects against Omicron infection in mice. Cell.

[2] Karim SSA, Karim QA (2021). Omicron SARS-CoV-2 variant: a new chapter in the COVID-19 pandemic. Lancet.

[3] McCallum M (2022). Structural basis of SARS-CoV-2 Omicron immune evasion and receptor engagement. Science.

[4] Halfmann PJ (2022). SARS-CoV-2 Omicron virus causes attenuated disease in mice and hamsters. Nature.

